# Comprehensive assessment of alterations in hand deformities over 11 years in patients with rheumatoid arthritis using cluster analysis and analysis of covariance

**DOI:** 10.1186/s13075-021-02448-4

**Published:** 2021-02-27

**Authors:** Shogo Toyama, Daisaku Tokunaga, Shinji Tsuchida, Rie Kushida, Ryo Oda, Yutaka Kawahito, Kenji Takahashi

**Affiliations:** 1 Department of Orthopaedics, Graduate School of Medical Science, Kyoto Prefectural University of Medicine, Kajii-cho 465, Kamigyo-ku, Kyoto, 602-8566 Japan; 2 Rehabilitation Unit, Marutamachi Rehabilitation Clinic, Kyoto, Japan; 3 Department of Inflammation and Immunology, Graduate School of Medical Science, Kyoto Prefectural University of Medicine, Kyoto, Japan

**Keywords:** Rheumatoid arthritis, Hand deformity, Swan-neck deformity, Boutonnière deformity, Cluster analysis

## Abstract

**Background:**

Although drug therapy for rheumatoid arthritis (RA) has recently improved, treating patients with established disease, whose hands have three major deformities (thumb deformity, finger deformities, and ulnar drift), remains a challenge. The underlying complex pathophysiology makes understanding these deformities difficult, and comprehensive assessment methods require accumulated skill with long learning curves. We aimed to establish a simpler composite method to understand the pathophysiology of and alterations in the hand deformities of patients with RA.

**Methods:**

We established a rheumatoid hand cohort in 2004 and clinically evaluated 134 hands (67 patients). We repeated the evaluations in 2009 (100 hands of 52 patients) and 2015 (63 hands of 37 patients) after case exclusion. Thumb deformities, finger deformities (swan-neck and boutonnière deformity), and ulnar drift were semi-quantitated and entered as parameters into a two-step cross-sectional cluster analysis for the data in 2004. The parameters in each cluster were plotted at each evaluation point. Two-way analysis of covariance was used to examine whether differences existed between evaluation points and clusters of deformity parameters.

**Results:**

Five clusters most appropriately described hand deformity: (i) cluster 1, minimal deformity; (ii) cluster 2, type 1 thumb deformity; (iii) cluster 3, thumb deformity and severe boutonnière deformity; (iv) cluster 4, type 2 or 3 thumb deformity and severe ulnar drift; and (v) cluster 5, thumb deformity and severe swan-neck deformity. Clusters 1 and 2 had higher function than cluster 5, and cluster 3 had moderate function. Clusters 1–4 had similar disease duration but showed different paths of deformity progression from disease onset. Clusters 1 and 2 represented conservative deformity parameters and clusters 3, 4, and 5 represented progressive deformity parameters. Over time, thumb deformity evolved into other types of deformities and swan-neck deformity worsened significantly.

**Conclusions:**

Our comprehensive analysis identified five deformity patterns and a progressive course in the rheumatoid hand. Knowledge of the characteristics of progressive deformity parameters may allow rheumatologists to more easily implement practical interventions and determine functional prognosis.

## Background

Rheumatoid hand, the term used to describe the characteristic deformities in the hands of patients with rheumatoid arthritis (RA), typically includes varying degrees of thumb deformity, finger deformities, and ulnar drift. Several studies have attempted to quantitate hand function using hand space and force-time curves to evaluate hand deformity [1, 2]. Swan-neck deformity, either alone or in combination with other deformities, has been reported to affect hand function to a greater degree than other deformities. However, it remains difficult to understand patients’ overall pathophysiological condition (deformity presence or absence, location and severity, and alterations over time) to determine the most relevant treatment options.

A study of early RA patients over a 10-year period showed that approximately 50% of hands exhibited combined deformity [3]. However, deformity severity was not described. Although the authors did not evaluate thumb deformity and did not assess severity of finger deformities, they demonstrated that deformities were present in regular patterns such as “ulnar drift combined with swan-neck deformity.” Thus, there may be specific developmental patterns of deformities in rheumatoid hands, including the thumb. Another study described deformity severity in patients with established RA over 5 years of observation and found that overall deformities worsened over time [4]. The authors evaluated thumb deformity using the Nalebuff classification system (type 1–6) [5], finger swan-neck deformity using the Nalebuff classification (type 1–4) [6], finger boutonnière deformity using the Nalebuff classification (stage 1–3), and ulnar drift using the authors’ own method [7], which quantitated drift by evaluating joint parameters in an extended cohort [8].

Based on these findings, we hypothesized that a semi-quantitative approach using type/stage and ulnar drift parameters would provide a comprehensive understanding of the rheumatoid hand. We thus aimed to perform a comprehensive assessment to clarify the natural course of hand deformity and function in patients with RA.

## Methods

### Patients

**Fig. 1:**
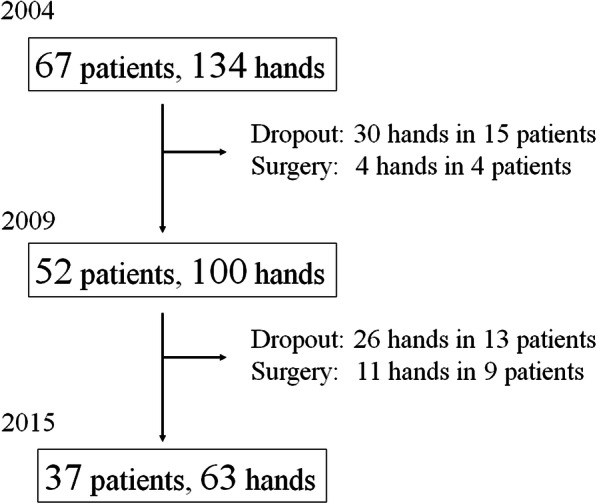
Flow diagram of the study. Patient registration in our rheumatoid hand cohort. Hands contralateral to the surgery side whose reconstruction surgery has performed were allowed to remain included in the cohort

Because biological agents were first approved for treatment of RA in Japan in 2003, data collection began in 2004. We enrolled outpatients with RA with any apparent hand deformities in either hand visiting our hospital. Among 83 patients who were included, 67 patients (134 hands) formed the cohort and participated in the follow-up survey. We collected serial data in 2009 and 2015 among patients who could be followed-up. Because of losses to follow-up (*n* = 7), death (*n* = 1), clinic change (*n* = 6), stroke (*n* = 1), and surgical treatment of the hands (*n* = 4, hands = 4), 52 patients (100 hands) were available for reevaluation in 2009. An additional six patients were lost to follow-up: six died, one developed severe dementia and was excluded from the cohort, and nine (11 hands) underwent hand surgery and were excluded. In 2015, we completed a final follow-up survey of 37 patients (two men and 35 women; 63 hands) (Fig. 1) [8].

At each evaluation, we evaluated hand deformities and performed functional assessments. Drug therapies were prescribed in accordance with globally used treatment guidelines (the guidelines/recommendations of the European League Against Rheumatism and the American College of Rheumatology changed several times during the follow-up period).

### Evaluation of hand deformity

Thumb deformity and swan-neck or boutonnière deformities in all four finger digits were assessed according to the Nalebuff classification of thumb deformity (type 1–6) [5] and the Nalebuff classifications of swan-neck deformity (type 1–4) and boutonnière deformity (stage 1–3) for the fingers [6, 7], respectively. Ulnar drift (total score 0–8) was assessed by assigning scores for four parameters of the metacarpophalangeal joints (deviation, subluxation, reduction, and bone destruction), with the score ranging from 0 to 2 for each parameter [8]. Hand deformities were evaluated by surgeons specializing in the rheumatoid hand.

### Evaluation of hand function

Patient-rated subjective indicators of unilateral hand function show improved performance for polyarticular diseases such as RA. Therefore, we adopted the Kapandji index as a measure of function at the outset of our study [9]. The Kapandji index evaluates finger extension (20 points), finger flexion (20 points), and thumb opposition (10 points), yielding a maximum score of 50 points. The Kapandji index can evaluate unilateral hand function within minutes, independent of the elbow and shoulder joints. Previous studies have used this functional evaluation method [10]. An occupational therapist and a specialist in hand therapy independently conducted the functional evaluation (Kapandji index).

### Data management and comprehensive assessment of hand deformity

The outcomes of clinical evaluations were managed independently by a hand surgeon and one of the study authors.

A comprehensive understanding of the rheumatoid hand, including characteristic deformities, was derived from cluster analysis of clinical parameters. The numerical values for thumb deformity (0–6) were entered as nominal variables. To our knowledge, no previous studies have compared hand function between deformity types. In real-word practice, type 3 deformities can occur directly with no pre-existing deformity, and there are many cases of deformity severity increasing in non-continuous fashion.

Finger deformities exist independently in all fingers with varying severities. The presence of swan-neck or boutonnière deformity also varies. Therefore, several parameters should be considered for each finger independently in the analysis (finger, type of deformity, and severity). Because the thumb has the most important functional role in both rheumatoid and normal hands [11, 12], we considered that parameters should be weighted to emphasize the impact of thumb deformity in the cluster analysis. Therefore, the scores for finger deformity according to the Nalebuff classification (swan-neck deformity: 0–4; boutonnière deformity: 0–3) were calculated separately for each finger We entered the values for these two scores as ordinal variables.

The ulnar drift score was an ordinal variable entered directly [8]. We performed two-step cluster analysis using the log likelihood ratio because one of the parameters was a nominal variable in the 2004 dataset. The number of clusters was determined according to silhouette measures of cohesion and separation as well as predictors of importance. The characteristics of each cluster were determined according to the distributions of the entered parameters.

### Alterations in hand deformity over time

To identify alterations over time in each cluster assigned as of 2004, the means of parameters were plotted for 2004, 2009, and 2015 for each cluster. Because disease duration impacts the degree of deformity, duration was used as a covariate in the analysis. Moreover, to examine whether differences existed between clusters and evaluation points, we conducted a two-way between-subject analysis of covariance (ANCOVA) with clusters and evaluation points as the independent variables and the swan-neck deformity score, the boutonnière deformity score, and the ulnar drift score as dependent variables. The post hoc Bonferroni’s test was used to correct for multiple comparisons. Another ANCOVA was conducted with the Kapandji index as the dependent variable to examine whether any differences were present in hand function.

## Results

### Patient demographics and drug therapy

Patient demographics are shown in Table 1. Disease activity generally improved over time despite increasing patient age. Only three patients were treated with biological agents in 2004, while seven and thirteen patients were treated with biological agents in 2009 and 2015, respectively.

**Table 1:** Demographics of patients in 2004, 2009 and 2015

		2004	2009	2015	*p* value
age	(year)	62.2	66.3	70.1	N/A
affected time	(year)	18.1	23.1	28.5	N/A
CRP	(mg/dl)	1.31	0.97	0.79	0.275
ESR	(mm/hr)	52.1	27.9*	34.7**	0.001
MMP-3	(ng/dl)	179.5	149.1	171.5	0.743
DAS28		*no data*	3.17	3	0.32

Numbers show the averages at each endpoint. Analysis of variance with post hoc Bonferroni test was used for multiple comparison

*CRP* C-reactive protein, *ESR* erythrocyte sedimentation rate, *MMP* matrix metalloprotease, *DAS* disease activity score

*: *p* = 0.001, *: *p* = 0.019

### Demographics of each cluster

The demographics and deformity scores for each cluster in 2004 are shown in Table 2. Cluster numbers were not automatically assigned by the software and were instead assigned in order of increasing degree of deformity. The characteristics of each cluster were as follows: (i) cluster 1: minimal deformity, minimal finger deformities and ulnar drift without thumb deformity; (ii) cluster 2: thumb type 1, type 1 thumb deformity and minimal finger deformities with ulnar drift; (iii) cluster 3: thumb and boutonnière deformity, type 1 or 6 thumb deformity and severe boutonnière deformity with ulnar drift; (iv) cluster 4: thumb type 2 or 3 and ulnar drift, type 2 or 3 thumb deformity with severe ulnar drift; and (v) cluster 5: thumb and severe swan-neck deformity, various types of thumb deformity and severe swan-neck deformity with ulnar drift.

**Table 2:** Patients demographics and parameter scores for rheumatoid hand deformities in each cluster

	Cluster 1	Cluster 2	Cluster 3	Cluster 4	Cluster 5
Patients’ demographics					
N	49	46	12	8	17
age (year)	64.0 (9.9)	61.2 (9.3)	62.5 (8.4)	51.3 (10.2)$	63.8 (7.6)**
disease duration (year)	16.2 (8.4)	17.1 (7.2)	19.7 (7.6)	17.4 (6.2)	25.5 (10.8)*, ***
Kapandji Index	34.4 (10.9)	35.8 (9.3)	30.5 (7.4)	25.8 (6.6)	22.5 (8.9)*, ***
Deformity parameters					
Swan-neck deformity	1.6 (0.3)	1.4 (0.2)	1.6 (0.6)	1.9 (0.5)	12.1 (0.8)^%^
Boutonnière deformity	1.2 (2.0)	0.2 (0.7)	5.3 (2.7)^#^	1.4 (2.7)	0.0 (0.0)
Ulnar drift	2.9 (2.7)	3.7 (2.6)	3.4 (1.1)	6.6 (1.4)^%^	3.6 (1.7)

Numbers express the mean (standard deviation). Analysis of variance with post-hoc Bonferroni was performed for multiple comparison

*: *p* < 0.05; cluster 1 and 5, **: *p* < 0.05; cluster 4 and 5, ***: *p* < 0.05; cluster 2 and 5, $: *p* < 0.05, cluster 1 and 4

%: *p* < 0.05, against all the other clusters, #: *p* < 0.05, against cluster 1, 2, and 4

**Fig. 2:**
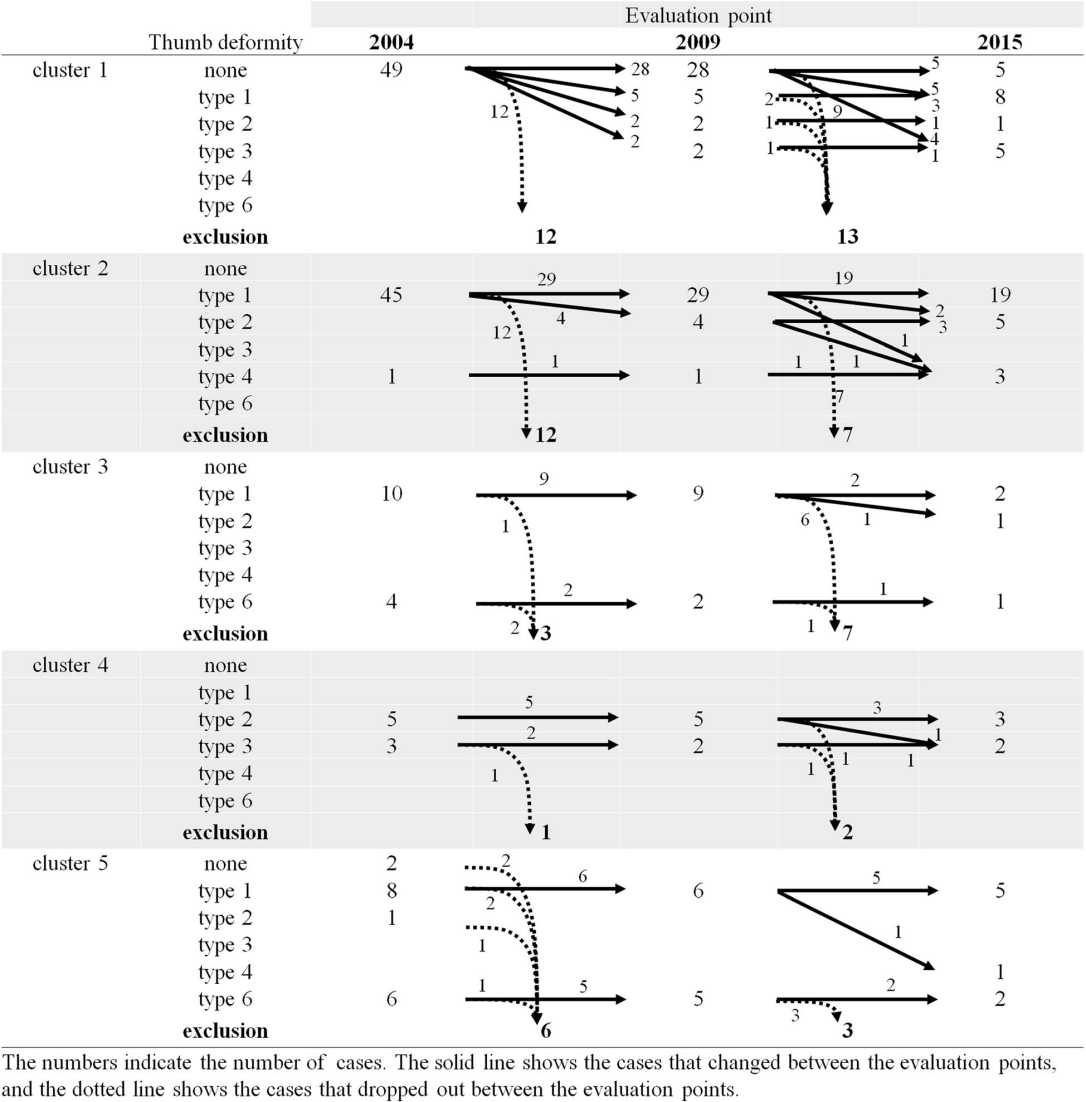
Thumb alterations in patients with rheumatoid arthritis. Progression of thumb deformity over time. Several specific patterns of deformity progression can be seen

The patients in cluster 4 were significantly younger but did not have shorter disease durations than patients in other clusters. Disease duration was significantly longer in cluster 5 patients. Patients in cluster 5 scored significantly lower on the Kapandji index, while those in cluster 4 showed a trend toward lower scores that was not statistically significant. Swan-neck and boutonnière deformity scores were higher in certain clusters (Table 2), while ulnar drift scores were moderately increased in clusters that were not otherwise significantly different from one another. Numbers of thumb deformities are shown in Fig. 2. In our cohort, no type 5 thumb deformities were observed at any evaluation point.

### Deformity alterations in each cluster over time

Alterations in thumb deformity are shown in Fig. 2. Of thumbs without deformity in cluster 1, 75.7% (28/37) at 5-year follow-up and 26.3% (5/19) at 10-year follow-up retained no deformity. Development of type 1, 2, and 3 deformities at follow-up occurred in other cluster 1 thumbs. In the other clusters, alterations of type 1 to type 2 deformities were often observed. In one case, there was an alteration from type 2 to type 3 deformity.

**Fig. 3:**
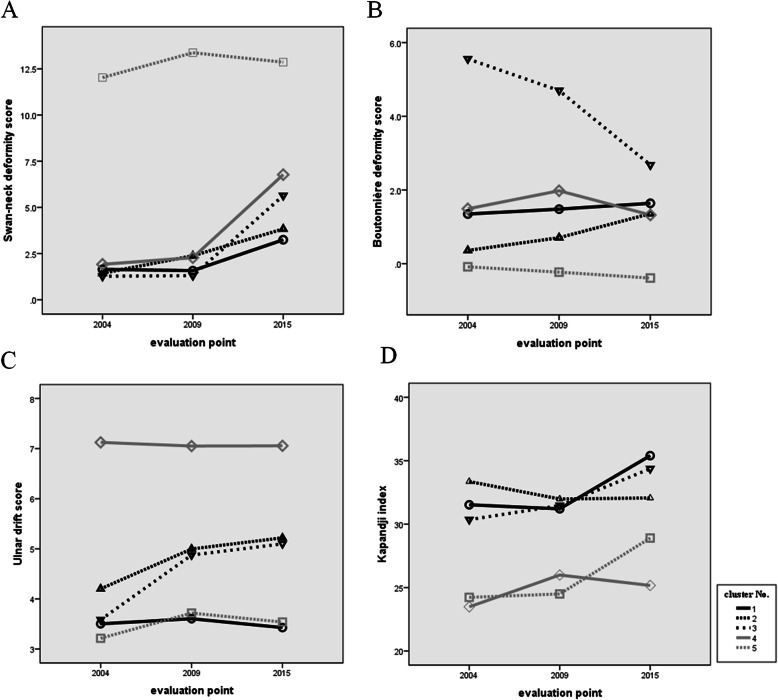
Trajectory plots of swan-neck deformity scores, boutonnière deformity scores, ulnar drift scores and the Kapandji index over time. Trajectory plots of swan-neck deformity scores (**a**), boutonnière deformity scores (**b**), ulnar drift scores (**c**), and the Kapandji index (**d**). Differences between clusters are shown. Functional impairment did not worsen during the 11 years of the study

Plots of swan-neck deformity scores, boutonnière deformity scores, and ulnar drift scores are shown in Fig. 3a–c. ANCOVA of swan-neck deformity scores showed there was no interaction between clusters and evaluation points. Multiple comparisons were performed because there was a significant main effect of cluster (*F* = 101.638, *p* < 0.001) and evaluation point (*F* = 13.827, *p* < 0.001). Estimates of the magnitudes of differences between clusters are shown in Table 3. Cluster 5 was significantly different from all other clusters, and there were also significant differences between 2004 and 2015 and between 2009 and 2015. For the boutonnière deformity score, there was no interaction between clusters and evaluation points. There was a significant main effect of cluster (*F* = 14.607, *p* < 0.001); however, the main effect of evaluation point (*F* = 0.457, *p* = 0.633) was not significant. In multiple comparisons, cluster 3 was significantly different compared with all other clusters, and there was also a significant difference between clusters 1 and 5. For the ulnar drift score, there was no interaction between clusters and evaluation points. There was a significant main effect of cluster (*F* = 10.927, *p* < 0.001), but the main effect of evaluation point (*F* = 0.970, *p* < 0.380) was not significant.

**Table 3:** Mean differences from multiple comparisons between clusters

		Cluster 2	Cluster 3	Cluster 4	Cluster 5
Swan-neck deformity	**cluster 1**	− 0.395	−0.583	−1.501	− 10.600*
	**cluster 2**		− 0.188	−1.106	− 10.205*
	**cluster 3**			−0.918	−10.017*
	**cluster 4**				−9.099*
Boutonnière deformity	**cluster 1**	0.681	−2.824*	−0.108	1.723*
	**cluster 2**		−3.505*	−0.789	1.043
	**cluster 3**			2.716*	4.547*
	**cluster 4**				1.832
Ulnar drift	**cluster 1**	−1.295*	−1.008	−3.565*	0.021
	**cluster 2**		0.286	−2.271*	1.316
	**cluster 3**			−2.557*	1.029
	**cluster 4**				3.586*
Kapandji index	**cluster 1**	0.242	0.647	7.832*	6.834*
	**cluster 2**		0.405	7.590*	6.592*
	**cluster 3**			7.185	6.187
	**cluster 4**				−0.998

The numbers express mean differences between clusters

Multiple comparisons were performed with post-hoc Bonferroni. *: *p* < 0.05

In multiple comparisons, cluster 4 was significantly different compared with all other clusters, and there was also a significant difference between clusters 1 and 2. These results can be interpreted as follows. Cluster 1 included hands that were originally minimally deformed, but developed thumb deformity and swan-neck deformity over time. Cluster 2 included the second least deformed hands after cluster 1, but a subset of cluster 2 hands showed progression from mainly type 1 thumb deformity to swan-neck deformity and ulnar deviation over time. Cluster 3 included a subset of hands with thumb deformities like those of cluster 2, but with a high degree of boutonnière deformity. Cluster 4 included hands with type 2 and 3 thumb deformities and a high degree of ulnar drift, and because these hands were already highly deformed, they showed little progression over time. Cluster 5 hands had the most severe thumb deformity (type 6) and a high degree of swan-neck deformity in roughly half of the hands.

### Functional alterations in each cluster over time

Changes in the Kapandji index over time are shown in Fig. 3d. ANCOVA showed no interaction between clusters and evaluation points, with a significant main effect of cluster (*F* = 10.707, *p* = 0.001) but not of evaluation point (*F* = 1.348, *p* = 0.273). In multiple comparisons, clusters 1 and 2 were significantly different compared with clusters 4 and 5 (Table 3).

## Discussion

In this study, rheumatoid hands were divided into clusters using parameters describing representative deformities. Alterations in hand deformity and function over time were compared with disease duration as a covariate. Although there have been reports of the incidence of hand deformities within 10 years of RA onset [2, 3], comprehensive assessments of the rheumatoid hand, including thumb deformity, and alterations in deformities over time have not been performed to the best of our knowledge.

Four of the five clusters had similar disease durations but showed wide variation in deformities. Thus, the period of symptomatic time affecting hand joints was the same, but cumulative damage during this period differed between the clusters. Cumulative disease activity in RA has been shown to affect the prognosis of the joint [13]; therefore, differences in the degree of deformity between clusters could have resulted from differences in the effectiveness of drug therapy from disease onset. However, because the transition of disease activity since onset could not be followed in each patient, cumulative disease activity was unknown. Large differences in hand phenotypes emerged over the approximately 17 years of disease duration covered by our study. The hand clusters identified in this study revealed a typical pattern of deformity progression. We also identified a subset of hands that developed few and mild deformities, which is good news from a clinical standpoint. Conversely, type 2 and 3 thumb deformities may be a sign to consider aggressive treatment, as these deformities are associated with strong functional impairment complicated by severe ulnar drift.

Generally, RA disease activity and duration are assessed on a person-by-person basis, whereas the prevalence of arthritis in both hands is not symmetrical. Therefore, we conducted a cluster analysis of 134 hands from 67 subjects in 2004, when the study was initiated. However, the cluster analysis does not account for correlation between the two hands of a single subject. If the clusters for both hands are the same, age and disease duration will be counted twice. However, choosing a representative hand for a single subject cannot be performed without bias. Therefore, in this study, both hands were treated independently for analysis. Roughly two thirds (46 of 67) of patients had the same clusters for both hands, while 21 patients had different clusters for each hand (data not shown). Thus, our strategy of analyzing each hand independently was appropriate and we believe our results are valuable, even if the background information for each cluster is not completely accurate.

We found that swan-neck deformity progressed significantly over time, whereas boutonnière deformity and ulnar drift were relatively stable. The lattermost result conflicts with reports showing that ulnar drift progresses, leading to decreased hand function over time [3, 14]. Trajectory plots indicated that the clusters identified in this study could be clearly divided into progressive and non-progressive, suggesting the possibility of a type II error because only 63 hands were available in 2015.

There was no significant difference between functional assessment scores over time; in fact, some groups showed a slight increase in mean scores. Overall, however, deformity parameters deteriorated, suggesting that factors other than deformity may have influenced our results. During the 11-year observation period, C-reactive protein levels and erythrocyte sedimentation rates improved. The number of biologics used increased from 3 (4.5%) in 2004, to 7 (13.5%) in 2009 and 13 (35.1%) in 2015. The effectiveness of biologics in improving rheumatoid hand function has been widely reported [15], and their increased use might have improved the scores in the present study.

The Nalebuff classification divides thumb deformities into six types by the initially affected joint and its appearance [16, 17]; type change over time is not considered. To our knowledge, no studies have compared hand function by thumb deformity type, and none have quantitated the impact of deformity type on hand function. Our results have some interesting implications. First, type 1 deformity appears to be the primary phenotype, and type 2 and 4 deformities are secondary phenotypes arising from type 1. Second, seven of the type 2 deformities observed in our study arose from type 1 deformities. Thus, a rheumatoid thumb with the “initially metacarpophalangeal” joint affected changed over time to a hand with the “initially carpometacarpal” joint affected. This finding may be explained by initial flexion contracture of the metacarpophalangeal joint with secondary carpometacarpal joint involvement. Third, type 3 deformity, with the “initially carpometacarpal” joint affected, occurred mainly in thumbs without deformity, except for in one case. Type 3 deformity would thus represent a primary phenotype like type 1. The underlying mechanisms influencing these phenotypic differences are still unknown. Our results may raise controversy regarding the underlying pathological mechanism of thumb deformities.

**Fig. 4:**
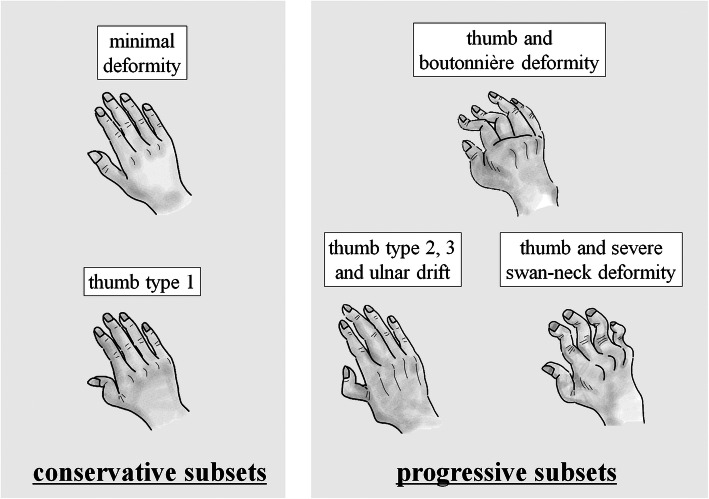
Paths of rheumatoid hand development. From the onset of rheumatoid arthritis, patterns were present in the development of hand deformities. There were two subsets in these patterns: a conservative subset with only type 1 thumb deformities, at most, and a progressive subset including hands that developed significant deformities. Multiple finger deformities, including swan-neck deformities, and type 2 and 3 thumb deformities could be a useful indicator of the progressive subset

Our comprehensive assessment has several implications for the development of hand deformities after the onset of RA (Fig. 4). The “minimal deformity” and “thumb type 1” clusters were considered conservative, while the “thumb and boutonnière deformity”, “thumb type 2, 3 and ulnar drift”, and “thumb and severe swan-neck deformity” clusters were considered progressive. Though we cannot indicate specific proportions, a majority of hands fit into the conservative subset, which is fortunate for patients and their clinical outcomes. Minimal finger deformity was considered to represent the conservative subset, whereas multiple deformities were characteristic of the progressive subset. It is difficult to confidently assign each hand to a subset. However, more attention should be paid to swan-neck deformity as this deformity progressed over our 11-year observation period. Type 2 and 3 thumb deformities may also be indicators of the progressive subset, complicated by severe ulnar drift. Similarly, type 6 thumb deformity is a clear indicator of progression.

Our study had several limitations. First, because the cohort was not followed from the onset of RA, we were unable to demonstrate how deformities developed in each subset. Understanding the order of occurrence of deformities might have enabled us to provide better treatments for these patients. Second, the cluster analysis assigned hands to each cluster retrospectively. Therefore, our clusters were explanatory in nature and cannot necessarily be applied to new single hands; additional studies are needed to assess the generalizability of these clusters. Third, the results of this study could have been more meaningful if the disabling effects of each type of thumb deformity were understood. A further comparison of thumb deformity in another cohort is warranted. Fourth, we used the Kapandji index as a functional evaluation. This index is usually used as a functional mobility measure and reflects functional impairment. Therefore, using an index that reflects unilateral disability, such as the Michigan Hand Outcomes Questionnaire [18], could be more reflective of disability in the rheumatoid hand. Unfortunately, we were unable to adopt these patient-reported outcome measures at the beginning of the study. Finally, the sample size was small and only eight patients were assigned to cluster 4. Therefore, the characteristics of this cluster were somewhat uncertain.

## Conclusions

Our comprehensive assessment of rheumatoid hand characteristics could be a useful tool for rheumatologists and physicians to better understand patients and impaired activities of daily living.

## Data Availability

Not applicable

## Abbreviations

RA: Rheumatoid arthritis
ANCOVA: Analysis of covariance
